# 

**Published:** 1887-04

**Authors:** 


### THE ILLINOIS STATE DENTAL SOCIETY.

The twenty-third annual meeting of the Illinois State Dental Society will be held at Jacksonville, beginning

Tuesday, May 10th and continuing four days. A full programme of scientific work is arranged for, including a large and instructive clinic. Dr. Black will give short lectures on micro-organisms with practical demonstration of their culture. All dentists will be cordially welcomed.

J. W. WASSALL,
*Secretary.*

---

### MISSOURI STATE DENTAL SOCIETY.

The Missouri State Dental Association will hold its twenty-third annual meeting at Kansas City, Mo., the third Tuesday in June, continuing in session four days, June 21st and 24th inclusive.

J. G. HARPER, D. D. S.,
*Recording Secretary.*

WILLIAM CONRAD, D. D. S,
*President.*

### *To Readers and Correspondents!*

Communications intended for publication, and exchange journals and papers should be addressed to the EDITOR OF THE AMERICAN JOURNAL OF DENTAL SCIENCE, 845 N. Eutaw St., Baltimore, Md. All letters relating to the business of the JOURNAL, or containing cash remittances, to be directed to Messrs. SNOWDEN & COWMAN. Articles for publication should be received by the editor at least three weeks previous to the first month on which the number in which it is designed for them to appear, is issued.

☞ The AMERICAN JOURNAL OF DENTAL SCIENCE will contain fifty pages of reading matter in each number, making a volume of about *Six Hundred* pages,

EXCLUSIVE OF ADVERTISEMENTS.

